# Cross-reactive Neutralizing Antibody Responses to Enterovirus 71 Infections in Young Children: Implications for Vaccine Development

**DOI:** 10.1371/journal.pntd.0002067

**Published:** 2013-02-14

**Authors:** Mei-Liang Huang, Pai-Shan Chiang, Min-Yuan Chia, Shu-Ting Luo, Luan-Yin Chang, Tzou-Yien Lin, Mei-Shang Ho, Min-Shi Lee

**Affiliations:** 1 National Health Research Institutes (NHRI), Zhunan, Taiwan; 2 National Taiwan University Hospital, Taipei, Taiwan; 3 Chang Gung Memorial Hospital (CGMH), Taoyuan, Taiwan; 4 Institute of Biomedical Sciences, Academia Sinica, Taipei, Taiwan; The George Washington University Medical Center, United States of America

## Abstract

**Background:**

Recently, enterovirus 71 (EV71) has caused life-threatening outbreaks involving neurological and cardiopulmonary complications in Asian children with unknown mechanism. EV71 has one single serotype but can be phylogenetically classified into 3 main genogroups (A, B and C) and 11 genotypes (A, B1∼B5 and C1∼C5). In Taiwan, nationwide EV71 epidemics with different predominant genotypes occurred in 1998 (C2), 2000–2001 (B4), 2004–2005 (C4), and 2008 (B5). In this study, sera were collected to measure cross-reactive neutralizing antibody titers against different genotypes.

**Methods:**

We collected historical sera from children who developed an EV71 infection in 1998, 2000, 2005, 2008, or 2010 and measured cross-reactive neutralizing antibody titers against all 11 EV71 genotypes. In addition, we aligned and compared the amino acid sequences of P1 proteins of the tested viruses.

**Results:**

Serology data showed that children infected with genogroups B and C consistently have lower neutralizing antibody titers against genogroup A (>4-fold difference). The sequence comparisons revealed that five amino acid signatures (N143D in VP2; K18R, H116Y, D167E, and S275A in VP1) are specific for genogroup A and may be related to the observed antigenic variations.

**Conclusions:**

This study documented antigenic variations among different EV71 genogroups and identified potential immunodominant amino acid positions. Enterovirus surveillance and vaccine development should monitor these positions.

## Author Summary

Recently, enterovirus 71 (EV71) has caused life-threatening outbreaks in tropical Asia. EV71 has one single serotype but can be phylogenetically classified into 3 main genogroups and 11 genotypes (A, B1∼B5 and C1∼C5). In Taiwan, nationwide EV71 epidemics with different predominant genotypes occurred in 1998(C2), 2000–2001(B4), 2004–2005(C4), and 2008(B5). In this study, historical sera from children infected with these 4 genotypes were collected to measure cross-reactive neutralizing antibody titers against 11 genotypes. In addition, amino acid sequences of P1 proteins of the tested viruses were compared. Serology data showed that children infected with genogroup B and C consistently have lower neutralizing antibody titers against genogroup A (>4-fold difference). Antigenic variations between genogroup B and C could be detected but did not have a clear pattern. Five amino acid signatures are specific for genogroup A and may be related to the observed antigenic variations. Vaccine development should monitor the antigenic and genetic variations to select vaccine strains.

## Introduction

Human enteroviruses include over 100 serotypes and usually cause self-limited infections, except polioviruses and enterovirus 71 (EV71) which frequently involve neurological complications [1], [2]. Although EV71 was first described in 1969, a retrospective analysis shows that this virus circulated in the Netherlands as early as 1963 [3]. Recent molecular evolution studies predicted that EV71 could have emerged in the human population around 1941 [4]. Globally, two patterns of EV71 outbreaks have been reported: small-scale outbreaks with low mortality and large-scale outbreaks with high mortality. The latter pattern occurred in Bulgaria with 44 deaths in 1975, in Hungary with 45 deaths in 1978, in Malaysia with 29 deaths in 1997, in Taiwan with 78 deaths in 1998, in Singapore with 5 deaths in 2000, and recently in China with more than 100 deaths every year after 2007. Due to its tremendous impact on healthcare systems, development of EV71 vaccines is a national priority in some Asian countries [2].

EV71 has one single serotype as measured by hyperimmune animal antiserum but can be phylogenetically classified into 3 genogroups (A, B and C) and 11 main genotypes (A, B1∼B5 and C1∼C5) by analyzing the most variable capsid protein sequences (VP1) [1]. Recently, one new genogroup was only detected in India [5]. Genogroup A viruses were isolated in 1970 in the United States and were not detected globally again until 2008. In an investigation of a HFMD outbreak in central China in 2008, Yu *et al* identified five EV71 isolates which were closely related to genotype A based on analysis of the VP1 gene [6]. In contrast, genogroups B and C are widely circulating in the world with different evolution patterns [7], [8]. Interestingly, genogroup replacements of EV71 have been well documented in Taiwan and Malaysia [1], [2]. In Taiwan, nationwide EV71 epidemics with different predominant genotypes occurred in 1998 (C2), 2000–2001 (B4), 2004–2005 (C4), and 2008 (B5) [9]–[11]. In this study, sera from EV71-infected children were collected to measure cross-reactive neutralizing antibody titers against different genotypes, which are critical to understand the drivers of genogroup replacement and viral diversity, and for selection of vaccine strains.

## Materials and Methods

### Ethics statement

Institutional review board approvals were obtained from Chang Gung Memorial Hospital, and National Taiwan University following the Helsinki Declaration. Written informed consents were obtained from parents/guardians on behalf of all child participants.

### Sera

To avoid confounding effects of EV71 re-infections, historical sera were collected from young children who were under 5 years of age and infected with different EV71 genotypes in 1998 (genotype C2, 10 sera), 2000 (genotype B4, 5 sera), 2005 (genotype C4, 2 sera), 2008 (genotype B5, 5 sera), or 2010 (genotype C4, 3 sera) [10], [12]–[14]. These sera were used to measure cross-reactive neutralizing antibody titers against all 11 EV71 genotypes.

### Virus

Twelve strains of the 11 EV71 genotypes were used in the study, including two genotype C4 viruses which were isolated in 2005 and 2008, respectively. Eight of these twelve viruses were isolated in Taiwan and the other four viruses (genotype A, B2, B3 and C3) had not circulated in Taiwan (Table 1). All viruses were amplified in rhabdomyosarcoma (RD) cells using Dulbecco's Minimum Essential Medium (DMEM) containing fetal bovine serum 2% v/v and penicillin/streptomycin. Virus titers (50% tissue culture infectious doses, TCID_50_) were determined in RD cells using the Reed-Muench method.

**Table 1:** EV71 virus strains used for detecting cross neutralizing antibodies in this study.

Genotype	Virus name	Year of isolation	country of isolation	Abbreviate	Accession No.
A	A-U22521_BrCr	1970	USA	A-70	JN874547
B1	242-TW-86	1986	Taiwan	B1-86	JN874548
B2	86-11316	1986	Netherlands	B2-86	JN874549
B3	SK-EV006	1997	Malaysian	B3-97	JN874550
B4	E59P2-TW-02	2002	Taiwan	B4-02	JN874551
B5	NHRI141-TW-08	2008	Taiwan	B5-08	JN874552
C1	TW-4215-1998	1998	Taiwan	C1-98	JN874553
C2	Tainan/5746/98	1998	Taiwan	C2-98	JN874554
C3	001-KOR-00	2000	Korea	C3-00	JN874555
C4	70516TW-08	2008	Taiwan	C4-08	JN874556
C4	N1862TW-05	2005	Taiwan	C4-05	JN874557
C5	1575TW-07	2007	Taiwan	C5-07	JN874558

### Sequence analysis

The P1 region of the EV71 genome encodes four capsid proteins including VP1, VP2, VP3 and VP4 proteins, which are involved in the induction of immune response and the infection of cells [15]–[17]. Therefore, the P1 regions of 11 EV71 genotypes were sequenced to identify correlations between genetic and antigenic variations. Viral genomic RNA was extracted from 140 µL of virus culture isolates using a QIAmp Viral RNA kit (Qiagen, USA) according to the manufacturer's instructions. cDNA of EV71 was synthesis by SuperScript II Reverse Transcriptase (Invitrogen, USA). PCR reactions were performed by specific primers and KAPA HiFi DNA Polymerase (Kapa Biosystems, USA). Primers used in this study are listed in Supporting Table S1. Nucleotide sequences of P1 regions (2586 bp) were aligned and analyzed by the Mega 4 software (Molecular Evolutionary Genetics Analysis software version 4.0) [18]. Phylogenetic trees were constructed by the neighbor-joining method using the Maximum Composite Likelihood method and the prototype CA16 strain (CA16/G-10) as the outgroup virus. The reliability of the tree was estimated using 1,000 bootstrap replications. Nucleotide sequences analyzed in this study have been submitted to GenBank.

### Serologic assays

Laboratory methods for measuring EV71 serum neutralizing antibody titers followed standard protocols [19], [20]. Briefly, 50 µL of two-fold serially diluted sera and virus working solution containing 100 TCID_50_ of EV71 were mixed on 96-well microplates and incubated with RD cells. A cytopathic effect was observed in an inverted microscope after an incubation period of 4–5 days. Each serum dilution includes three replicates and the neutralization titers were read as the highest dilution that could result in a reduction of the cytopathic effect in at least two of three replicate wells. Each test sample was run simultaneously with cell control, positive serum control, and virus back titration. If the ratios of neutralizing antibody titers between different genotypes were greater than 4, we measured neutralizing antibodies titers at least three times to confirm the accuracy of tests.

### Antigenic cartography

Large tabular serological data are hard to summarize and are recently analyzed using antigenic cartography (i.e., antigenic map) [11], [21], [22]. Briefly, antigenic cartography is a way to visualize and increase the resolution of serological data, such as neutralization data. In an antigenic map, the distance between a serum point *S* and antigen point *A* corresponds to the difference between the log_2_ of the maximum titer observed for serum *S* against any antigen and the log_2_ of the titer for serum *S* and antigen *A*. Thus, each titer in a neutralization assay table can be thought of as specifying a target distance for the points in an antigenic map. Modified multidimensional scaling methods are used to arrange the antigen and serum points in an antigenic map to best satisfy the target distances specified by the neutralization data. The result is a map in which the distance between points represents antigenic distance as measured by the binding assay [11]. In this study, an antigenic map was generated using a web-based analytic tool [22].

### Statistical analysis

Neutralizing antibody titers were log transformed to calculate the geometric mean titers (GMTs), and their 95% confidence intervals (95% CI). The GMTs of cross-reactive neutralizing antibody titers were further used to generate an antigenic map using a web-based analytical tool [22]. The relative positions of strains and antisera were adjusted such that the distances between strains and antisera in the map represent the corresponding ratios between homologous and heterologous neutralizing antibody titers. Differences between homologous and heterologous neutralizing antibody titers were tested for statistical significance by the nonparametric tests (NPAR1WAY Procedure) using SAS software (SAS Institutes, Cary, NC).

### Accession number

Nucleotide sequences analyzed in this study have been submitted to GenBank (accession numbers JN874547–JN874558).

## Results

### Cross-reactivity between EV71 genogroups

Twenty-five sera were collected from 25 young children who were infected with EV71 genotype C2, B4, C4, B5, and C4 in 1998, 2000, 2005, 2008 and 2010, respectively. Cross-reactive neutralizing antibody titers against 11 EV71 genotypes are shown in Table 2. Overall, all EV71-infected children had detectable neutralizing antibody titers against 11 EV71 genotypes. Interestingly, homologous neutralizing antibodies titers were not always higher than heterologous neutralizing antibody titers.

**Table 2:** Cross-reactive neutralizing antibody titers against 11 EV71 genotypes in children infected by EV71 C2, C4, B4 and B5 genotypes.

Year of collection (genotype)	ID	Age	A-70	B1-86	B2-86	B3-97	B4-02	B5-08	C1-98	C2-98	C3-00	C4-05	C4-08	C5-07
1998(C2)	1	1.7	64	256	512	256	256	256	256	256	256	512	1024	256
	2	4.1	32	64	128	64	32	64	64	32	32	64	128	64
	3	3.1	64	256	256	256	512	256	256	256	256	256	256	256
	4	0.8	64	512	512	256	256	256	1024	256	256	256	512	512
1998(C2)	5	1.5	2048	8192	8192	4096	4096	4096	16384	8192	4096	8192	8192	4096
	6	0.8	256	1024	1024	1024	512	512	2048	1024	1024	1024	256	1024
	7	0.6	128	256	256	256	256	256	256	256	256	128	256	256
	8	0.8	128	1024	512	1024	512	512	256	256	512	512	1024	512
	9	3.8	128	2048	256	2048	512	1024	1024	1024	1024	1024	1024	1024
	10	4.2	128	512	512	512	512	256	512	256	256	512	512	512
2005(C4)	11	1.1	256	512	1024	1024	1024	1024	512	256	512	1024	2048	512
	12	4.3	128	512	512	128	256	256	256	256	256	256	512	256
2010(C4)	13	4.4	128	256	512	512	512	512	128	1024	256	512	512	512
	14	1.4	1024	2048	2048	4096	4096	2048	2048	4096	2048	2048	8192	2048
	15	2.4	32	128	128	64	128	128	128	256	64	128	256	128
2000(B4)	16	2.8	64	256	512	512	128	256	256	128	128	256	512	128
	17	0.5	64	256	512	512	512	256	256	256	128	256	128	256
	18	3.1	32	128	512	256	256	256	128	128	128	128	64	128
	19	2.7	16	64	64	64	32	64	64	16	32	32	32	32
	20	1.3	32	128	128	128	512	128	64	64	256	64	64	64
2008(B5)	21	2.08	32	64	512	128	64	128	32	32	128	32	64	64
	22	2.00	64	128	256	256	64	256	32	128	128	64	32	128
	23	2.25	64	128	512	256	128	256	64	128	256	64	128	128
	24	2.08	64	128	512	256	64	128	128	128	256	64	64	128
	25	2.25	32	64	128	128	128	128	64	128	128	64	64	64

**Figure 1:**
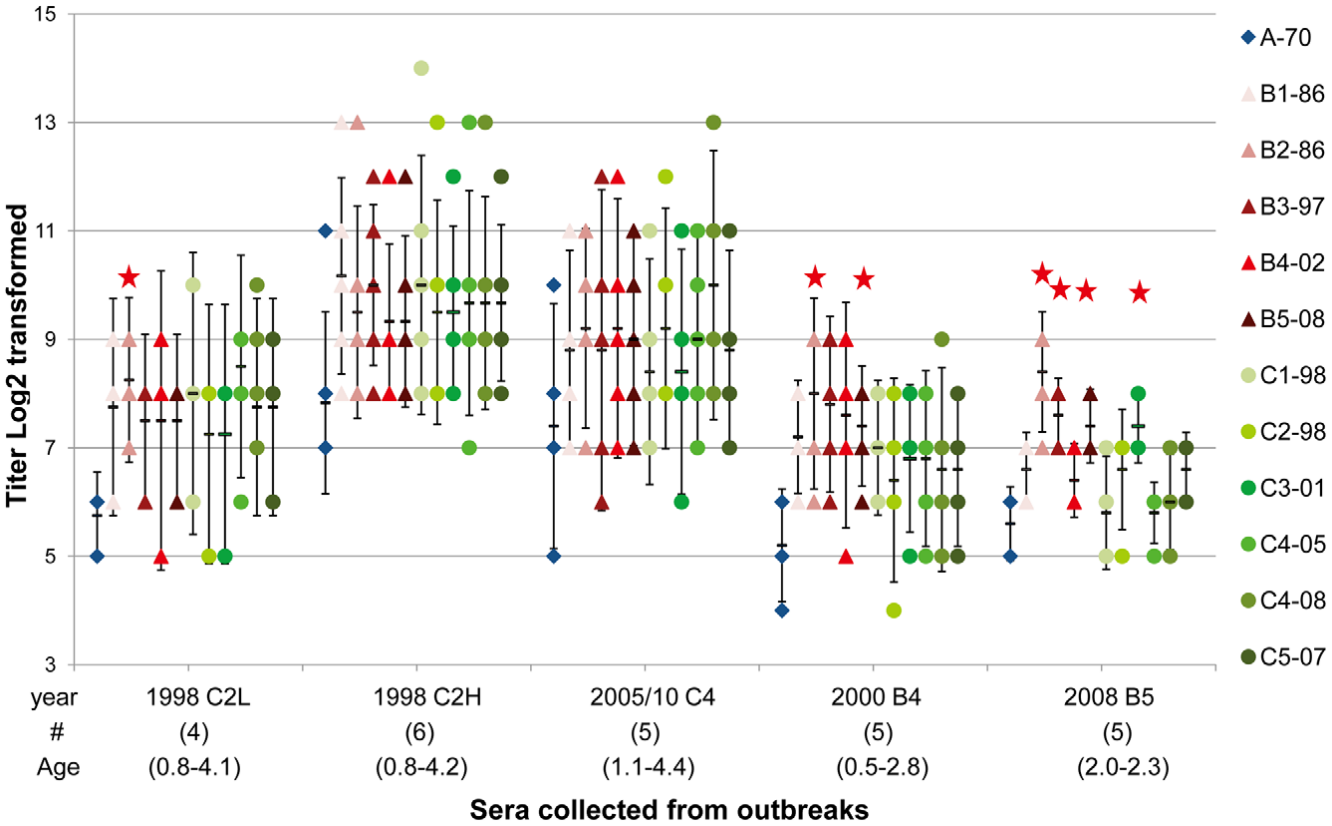
Serum neutralizing antibody titers against 11 EV71 genotypes (12 viruses) in young children. Sera were collected from young children infected with genotypes C2, C4, B4 and B5 viruses at different years. The dots indicate individual antibody titers and the bars indicate geometric mean titers (GMT) and their 95% confidence intervals. Serum neutralizing antibody titers against the homologous genotype (C2) in children infected in 1998 varied over 100-fold so they were grouped into two groups (low and high titers) for calculating GMT.

**Figure 2:**
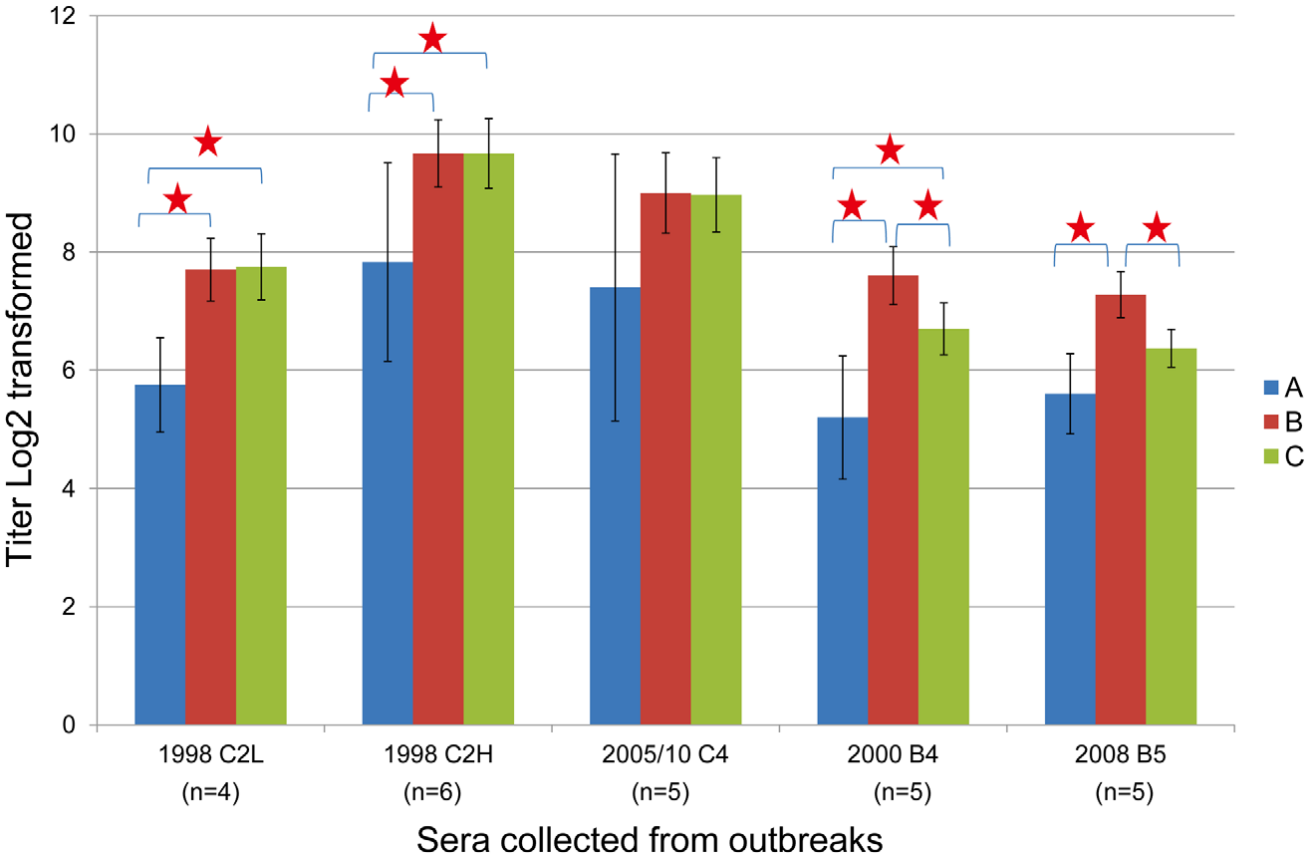
Distribution of serum neutralizing antibodies against three EV71 genogroups in young children. Sera were collected from young children infected with genotype C2, C4, B4 and B5 viruses at different years. Antibody titers against different genotypes within the same genogroup were used to calculate geometric mean titers for each genogroup. The bars indicate 95% confidence intervals of geometric mean titers (GMT). Serum neutralizing antibody titers against the homologous genotype (C2) in children infected in 1998 varied over 100-fold so they were grouped into two groups (low and high titers) for calculating GMT.

**Figure 3:**
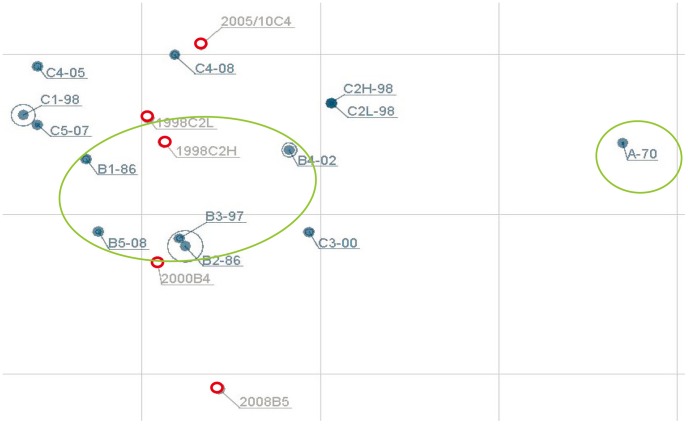
Antigenic map generated using serum cross-reactive EV71 neutralizing antibody titers presented in Figure 1. The relative positions of strains (black) and antisera (red) were adjusted such that the distances between strains and antisera in the map represent the corresponding ratios between homologous and heterologous neutralizing antibody titers. The spacing between grid lines is 1 unit of antigenic distance, corresponding to a 2-fold dilution of antiserum in the neutralization assay.

As shown in Table 2, serum neutralizing antibody titers against the homologous genotype (C2) in children infected in 1998 varied over 100-fold and they were grouped into two groups (low and high titers) for further analysis. In addition, children infected in 2005 and 2010 were merged for further analysis because they were all infected with genotype C4. GMTs of neutralizing antibody titers against 11 genotypes are shown in Figure 1. Overall, children infected with genotype C2, C4, B4 and B5 had lower GMTs (>4-fold difference) against genotype A than other genotypes. In contrast, antigenic variations between genogroup B and C did not have a clear pattern. We further merged neutralizing antibody titers against different genotypes within the same genogroup to calculate GMT for further comparisons. As shown in Figure 2, children infected with genotype C2 and C4 had similar GMT against genogroup B and C but children infected with B4 and B5 had higher GMTs against genogroup B than against genogroup C. We further constructed the antigenic map using GMT of cross-reactive neutralizing titers presented in Figure 1. Overall, genotypes in genogroup B and C clustered together and genotype A was found to be outside of the cluster (Figure 3).

### Sequence analysis of EV71 genotypes

To investigate the correlation between genetic and antigenic variations of EV71 genotypes, nucleotide and deduced amino acid sequences of P1 regions of 11 EV71 genotypes (12 viruses) were analyzed. Pairwise comparisons of P1 regions have shown that the nucleotide (amino acid) differences within EV71 genogroup were 0.049∼0.151 (0.005∼0.015) for Genogroup B and 0.042∼0.135 (0.005∼0.013) for Genogroup C and the nucleotide (amino acid) differences were 0.209∼0.224 (0.02∼0.026) between Genogroup A and B, 0.21∼0.235 (0.018∼0.024) between Genogroup A and C, and 0.188∼0.228 (0.021∼0.032) between Genogroup B and C (Table 3). Overall, the nucleotide differences in the P1 region within genogroup were much lower than that between genogroups (0.042∼0.151 vs. 0.188∼0.235) but the differences in amino acid sequences were not as abundant as found in nucleotide sequences (0.005∼0.015 vs. 0.018∼0.032) (Supporting Table S2). Genetic variations in VP1, VP2, VP3 and VP4 genes were also analyzed (Supporting Table S2). Interestingly, nucleotide differences in VP1∼VP4 were similar but no amino acid differences were observed in VP4 gene, which may exclude influence of VP4 on antigenic evolution of EV71.

**Table 3:** Pairwise nucleotide (lower left) and amino-acid (upper right) sequence differences between P1 genes of 12 EV71 viruses.

Virus ID		V1	V2	V3	V4	V5	V6	V7	V8	V9	V10	V11	V12
V1	A-BrCr-USA-70	-	0.023	0.023	0.021	0.026	0.02	0.024	0.02	0.019	0.018	0.02	0.021
V2	B1-242-TW-86	0.22	-	0.015	0.015	0.02	0.014	0.031	0.03	0.029	0.027	0.03	0.031
V3	B2-316-NLD-86	0.209	0.103	-	0.007	0.011	0.007	0.026	0.027	0.024	0.025	0.025	0.029
V4	B3-006-MA-97	0.218	0.124	0.06	-	0.006	0.005	0.026	0.026	0.024	0.024	0.024	0.027
V5	B4-E59-TW-04	0.224	0.136	0.069	0.049	-	0.008	0.03	0.031	0.027	0.029	0.029	0.032
V6	B5-141-TW-08	0.216	0.151	0.101	0.085	0.072	-	0.024	0.025	0.021	0.023	0.023	0.026
V7	C1-215-TW-98	0.217	0.217	0.212	0.228	0.226	0.224	-	0.012	0.006	0.013	0.013	0.011
V8	C2-746-TW-98	0.21	0.202	0.208	0.221	0.226	0.213	0.108	-	0.006	0.008	0.007	0.008
V9	C3-001-KOR-00	0.215	0.203	0.21	0.22	0.213	0.212	0.111	0.09	-	0.009	0.009	0.005
V10	C4-862-TW-05	0.213	0.199	0.19	0.205	0.209	0.211	0.125	0.11	0.119	-	0.005	0.012
V11	C4-516-TW-08	0.21	0.204	0.188	0.204	0.207	0.214	0.118	0.111	0.124	0.042	-	0.012
V12	C5-575-TW-07	0.235	0.213	0.212	0.216	0.222	0.217	0.114	0.118	0.135	0.135	0.135	-

Data are shown in proportion.

**Figure 4:**
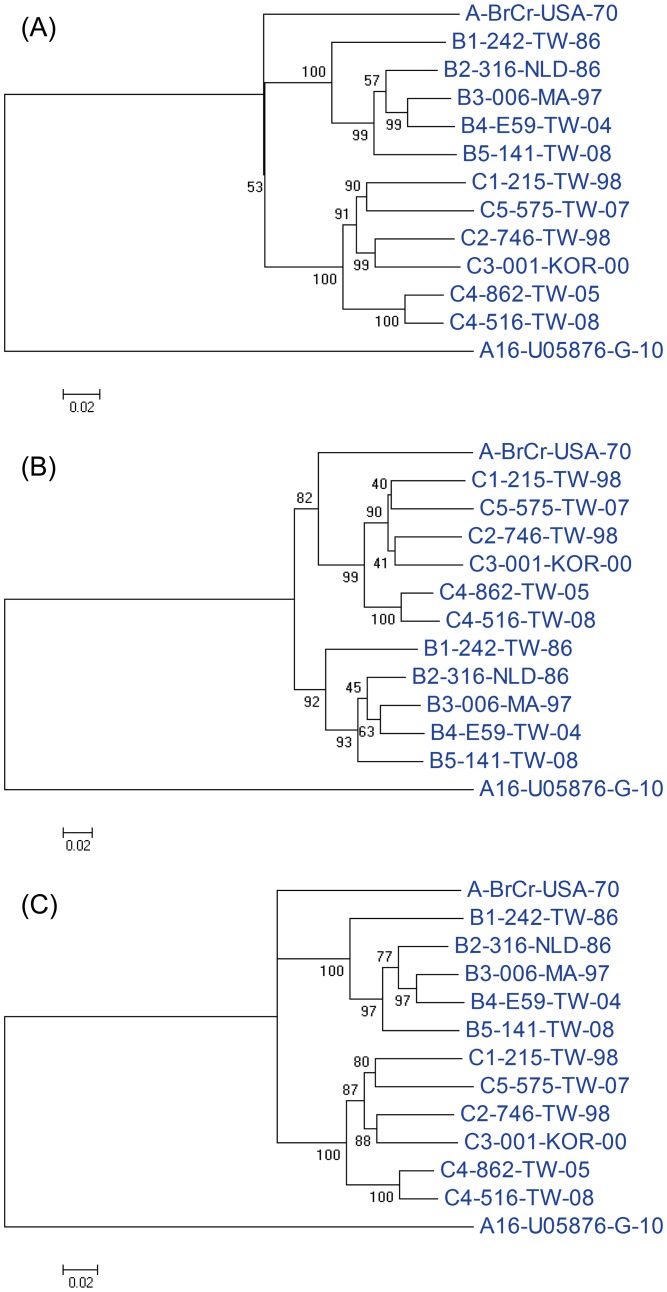
Phylogenetic analysis based on nucleotide sequences of EV71 strains compared in this study. The phylogenetic trees were generated using the P1 (A), VP1 (B) and VP1+VP3 (C) sequences. Virus identifications are shown in Table 1.

**Figure 5:**
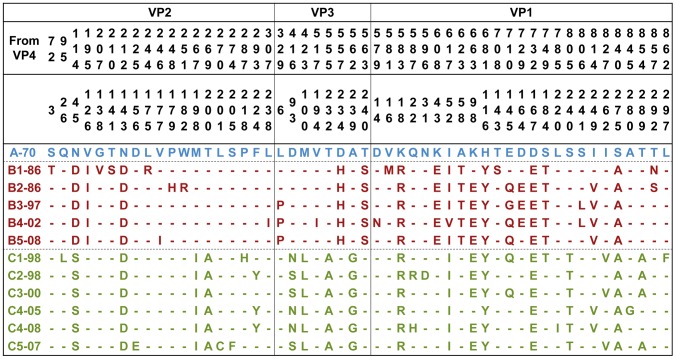
Alignment of P1 amino acid sequences of EV71 strains used for serological tests in this study. Virus identifications are shown in Table 1.

Phylogenetic analyses based on nucleotide sequences of the P1, VP1 and VP1+VP3 regions are shown in Figure 4. Overall, the phylogenetic trees generated using the P1 and VP1+VP3 regions indicated that genogroups B and C were distinct from the genotype A; however, the phylogenetic tree based on the VP1 region suggested that genogroup A is clustered with genogroup C. Overall, the phylogenetic relationship among the EV71 genotypes did not match with the antigenic relationship observed in this study. To further determine the amino acid differences related to the observed antigenic variations shown in Fig 1 and 3, the deduced amino acid sequences of P1 regions were aligned to reveal that five amino acid signatures (N143D in VP2; K18R, H116Y, D167E, and S275A in VP1) are specific for genogroup A and may be related to the observed antigenic variations (Figure 5).

## Discussion

EV71 has one single serotype as measured by hyperimmune animal antiserum but antigenic variations have been reported recently in human studies [9]–[11]. Using sera collected from young children with primary infection of genotype B5, two studies detected partial antigenic differences between genogroup B and C but not between viruses in the same genogroup (B5 and B4 viruses) [9], [10]. Kung *et al.* did not detect significant antigenic differences between genotypes B4 and C4 viruses using acute-phase sera from EV71 inpatients [23]. A serological survey in healthy Japanese children and adults detected partial antigenic differences between genotype B5 and A viruses but not among different genotypes in genogroup B and C that had previously circulated in Japan [24]. By constructing an antigenic map using 14 children sera, however, Huang et al. detected antigenic differences between genogroup B and C, and also between B5 and B4 viruses [11]. In a monkey study, Arita et al. [25] found that monkeys immunized with live-attenuated EV71 vaccine (genotype A) induced similar (<4-fold difference) antibody responses against genotype B1 but lower (≧4-fold difference) antibody responses against genotype B4, C2 and C4. In our study, we found that children infected with genotype C2, C4, B4 and B5 had lower GMTs (≧4-fold difference) against genotype A than other genotypes but antigenic variations between genogroup B and C did not have a clear pattern, which is different from the Huang study [11]. It is hard to compare different studies which had small sample size and employed different human sera and laboratory procedures, in particular the cell lines (RD cells vs. Vero cells) and virus strains used in the neutralization assay. A network to harmonize laboratory procedures including standard sera and viruses is required to make the comparison possible. Moreover, the clinical and epidemiological significance of the antigenic variation requires longitudinal serological studies to clarify.

Most clinical studies, including our study faced the limitation of small sample size due to the difficulty of collecting large amounts of serum samples from young children. Ideally, suitable animal models should be developed to generate a panel of antisera for monitoring EV71 antigenic variations, as ferrets served for influenza surveillance [26]. Representative EV71 clinical isolates could be selected for monitoring antigenic variations using the animal antisera. The clinical isolates with significant antigenic variations detected using animal antisera would be further evaluated using children post-infection sera.

Currently, five EV71 vaccine candidates are under evaluation in clinical trials, including three genogroup C viruses and two genogroup B viruses [27]. Based on the cross-reactive neutralizing antibody presented in the current study, genogroup B and C viruses are expected to induce protective neutralizing antibodies against genogroup B and C viruses but not genogroup A viruses. Interestingly, genogroup A viruses have disappeared for over 35 years but re-emerged in China in 2008. In an investigation of a HFMD outbreak in central China in 2008, Yu *et al* identified five EV71 isolates which were closely related to genotype A based on analysis of VP1 genes but these genogroup A viruses did not spread widely [6]. Reasons for the reemergence of genotype A in central China are not clear, and the full genomic sequences of the isolates should be performed to clarify the issue. Recently, novel genotype C2-like viruses were detected in Taiwan in 2008 and children infected with genotype C4, C5, B4 and B5 viruses had much lower (>100-fold) serum cross-reactive neutralizing antibody titers against the novel C2-like virus than against the homologous viruses. Interestingly, these novel C2-like viruses were recombinants of genotype C2 and B3 viruses but they did not spread widely [9]. Based on historical poliovirus studies, immunodominant neutralizing epitopes mainly locate on VP1 and VP2 proteins. Recently, binding sites of two EV71 mice neutralizing monoclonal antibodies were identified using synthetic peptide technology to locate at amino acid position 211–225 of VP1 protein and amino acid position 136–150 of VP2 protein, respectively [16]. The importance of these linear epitopes in the human immune response is not clear. In the current study, we combined human serological data and viral genetic sequence data to identify five amino acid positions (4 on VP1 protein and 1 on VP2 protein) related to antigenic variations. Only one of these five positions (VP2-143) was also identified in the mice monoclonal antibody studies. The clinical significance of these five positions needs to be verified using reverse genetics to generate mutant viruses. Recently, the 3-dimensional structures of EV71 capsid proteins have been published [28], [29]. Structural studies elucidating interaction between EV71 capsid proteins and neutralizing antibodies will help understand the mechanism of vaccine-induced immunity and design better vaccines.

Traditionally, the phylogenetic relationship of EV71 genotypes has been widely analyzed using VP1 nucleotide sequences [1] . Interestingly, a recent study found that the VP1-based phylogenetic tree is not similar to the complete genome-based phylogenetic tree [7]. Our study also found that the phylogenetic trees based on VP1 and P1 nucleotide sequences differ slightly. Specifically, genogroup A is close to genogroup C in the VP1-based phylogenetic tree but this relationship was not found in the P1-based phylogenetic trees. It is well known that enteroviruses including EV71 frequently recombine at the junction of structural (P1) and non-structural (P2 or P3) genes [8], [30]. Therefore, the P1 gene is suitable for phylogenetic analysis but the complete genome is required for detection of gene recombination. However, the P1 gene (about 3000 nucleotides) is much larger than the VP1 gene (about 890 nucleotides) and the P1 gene may not be readily available. The combined VP1+VP3 gene (about 1600 nucleotides) is much shorter than the P1 gene but could generate a similar phylogenetic tree to that based on the P1 gene. Overall, the VP1 gene is good enough for defining genotypes of genogroup B and C viruses, but it would be better to analyze the phylogenetic relationship between genogroup A viruses and other genogroup viruses based on the VP1+VP3 or P1 genes.

From an evolutionary perspective, a recent analysis of 628 EV71 VP1 sequences estimated that EV71 emerged in the human population around 1941 and evolved more quickly in the past 20 years [4]. It is unclear why EV71 has evolved more quickly in the past 20 years. Recombination, being a common occurrence among enteroviruses, might be the likely explanation for the emergence of EV71, but it would require full genome analysis to better understand the mechanism of EV71 evolution, which is critical to long-term success of EV71 vaccination programs.

## Supporting Information

Table S1 Primers used for PCR & sequence analysis.
(DOCX)

Table S2 Range of pairwise nucleotide and amino acid differences within and between EV71 genogroups.
(DOCX)

Text S1 STROBE checklist.
(DOC)
