# Supplementary material for: Cross-reactive Neutralizing Antibody Responses to Enterovirus 71 Infections in Young Children: Implications for Vaccine Development
Source: PLoS Negl Trop Dis. 2013 Feb 14;7(2):e2067. doi: 10.1371/journal.pntd.0002067 (PMC3573098; doi:10.1371/journal.pntd.0002067)
Supplement: Table S1 — Primers used for PCR & sequence analysis. (DOCX) [file pntd.0002067.s001.docx]

Table S1. Primers used for PCR & sequence analysis
