# Supplementary material for: Cross-reactive Neutralizing Antibody Responses to Enterovirus 71 Infections in Young Children: Implications for Vaccine Development
Source: PLoS Negl Trop Dis. 2013 Feb 14;7(2):e2067. doi: 10.1371/journal.pntd.0002067 (PMC3573098; doi:10.1371/journal.pntd.0002067)
Supplement: Table S2 — Range of pairwise nucleotide and amino acid differences within and between EV71 genogroups. (DOCX) [file pntd.0002067.s002.docx]

Table S2. Range of pairwise nucleotide and amino acid differences within and between EV71 genogroups

| Gene | Nucleotide | | Amino Acid | |
| --- | --- | --- | --- | --- |
|  | Within genogroup | Between genogroup | Within genogroup | Between genogroup |
| P1 | 0.042~0.151 | 0.188~0.235 | 0.005~0.015 | 0.018~0.032 |
| VP1 | 0.050~0.160 | 0.172~0.238 | 0.004~0.036 | 0.025~0.051 |
| VP2 | 0.044~0.156 | 0.172~0.241 | 0~0.024 | 0.012~0.037 |
| VP3 | 0.034~0.149 | 0.202~0.248 | 0~0.008 | 0.008~0.034 |
| VP4 | 0.030~0.184 | 0.181~0.264 | 0~0 | 0~0 |

Data are shown in proportion.
