# Supplementary material for: Cross-reactive Neutralizing Antibody Responses to Enterovirus 71 Infections in Young Children: Implications for Vaccine Development
Source: PLoS Negl Trop Dis. 2013 Feb 14;7(2):e2067. doi: 10.1371/journal.pntd.0002067 (PMC3573098; doi:10.1371/journal.pntd.0002067)
Supplement: Text S1 — STROBE checklist. (DOC) [file pntd.0002067.s003.doc]

STROBE Statement—Checklist of items that should be included in reports of ***cross-sectional studies***

|  | Item No | Recommendation |
| --- | --- | --- |
| **Title and abstract** | 1 | (*a*) Indicate the study’s design with a commonly used term in the title or the abstract  **Cross-reactive Neutralizing Antibody Responses to…** |
|  |  | (*b*) Provide in the abstract an informative and balanced summary of what was done and what was found  We collected historical sera from children who developed an EV71 infection in 1998, 2000, 2005, 2008, or 2010 and measured cross-reactive neutralizing antibody…  This study documented antigenic variations among different EV71 genogroups and identified potential immunodominant amino acid positions. |
| Introduction | | |
| Background/rationale | 2 | Explain the scientific background and rationale for the investigation being reported  EV71 has single serotype as measured by hyperimmune animal antiserum but can be phylogenetically classified into 3 genogroups (A, B and C) and 11 genotypes (A, B1~B5 and C1~C5) by analyzing the most variable capsid protein sequences (VP1)…  In Taiwan, nationwide EV71 epidemics with different predominant genotypes occurred in 1998 (C2), 2000–2001 (B4), 2004–2005 (C4), and 2008 (B5)… |
| Objectives | 3 | State specific objectives, including any prespecified hypotheses  In this study, sera from EV71-infected children were collected to measure cross-reactive neutralizing antibody titers against different genotypes, which are critical to understand the drivers of genogroup replacement and viral diversity and for selection of vaccine strains |
| Methods | | |
| Study design | 4 | Present key elements of study design early in the paper  historical sera were collected from young children who were under 5 years of age and infected with different EV71 genotypes to measure cross-reactive neutralizing antibody titers against all 11 EV71 genotypes |
| Setting | 5 | Describe the setting, locations, and relevant dates, including periods of recruitment, exposure, follow-up, and data collection  historical sera were collected from young children who were under 5 years of age and infected with different EV71 genotypes in 1998 (genotype C2, 10 sera), 2000 (genotype B4, 5 sera), 2005 (genotype C4, 2 sera), 2008 (genotype B5, 5 sera), or 2010 (genotype C4, 3 sera) |
| Participants | 6 | (*a*) Give the eligibility criteria, and the sources and methods of selection of participants  under 5 years of age and infected with different EV71 genotypes |
| Variables | 7 | Clearly define all outcomes, exposures, predictors, potential confounders, and effect modifiers. Give diagnostic criteria, if applicable  Outcomes: cross-reactive neutralizing antibody titers against all 11 EV71 genotypes,  Exposures: infected with different EV71 genotypes |
| Data sources/ measurement | 8* | For each variable of interest, give sources of data and details of methods of assessment (measurement). Describe comparability of assessment methods if there is more than one group  to measure cross-reactive neutralizing antibody titers against …  11 EV71 genotypes were used in the study…  Neutralizing antibody titers were log transformed to calculate the geometric mean titers (GMTs), and their 95% confidence intervals (95% CI). The GMTs of cross-reactive neutralizing antibody titers were further used to generate an antigenic map using a web-based analytical tool. The relative positions of strains and antisera were adjusted such that the distances between strains and antisera in the map represent the corresponding ratios between homologous and heterologous neutralizing antibody titers. |
| Bias | 9 | Describe any efforts to address potential sources of bias  Each test sample was run simultaneously with cell control, positive serum control, and virus back titration. If the ratios of neutralizing antibody titers between different genotypes are greater than 4, we measure neutralizing antibodies titers at least 3 times to confirm the accuracy of tests. |
| Study size | 10 | Explain how the study size was arrived at  This is an exploratory study and we have collected historical sera as many as possible. |
| Quantitative variables | 11 | Explain how quantitative variables were handled in the analyses. If applicable, describe which groupings were chosen and why  two-fold serially diluted sera… |
| Statistical methods | 12 | (*a*) Describe all statistical methods, including those used to control for confounding  Differences between homologous and heterologous neutralizing antibody titers were tested for statistical significance by the nonparametric tests (NPAR1WAY Procedure)… |
|  |  | (*b*) Describe any methods used to examine subgroups and interactions  **-** |
|  |  | (*c*) Explain how missing data were addressed  NO missing data |
|  |  | (*d*) If applicable, describe analytical methods taking account of sampling strategy  **-not applicable** |
|  |  | (*e*) Describe any sensitivity analyses  **-Not applicable** |
| Results | | |
| Participants | 13* | (a) Report numbers of individuals at each stage of study—eg numbers potentially eligible, examined for eligibility, confirmed eligible, included in the study, completing follow-up, and analysed  numbers of individuals at each stage of study: not applicable |
|  |  | (b) Give reasons for non-participation at each stage **–**not applicable |
|  |  | (c) Consider use of a flow diagram **– not applicable** |
| Descriptive data | 14* | (a) Give characteristics of study participants (eg demographic, clinical, social) and information on exposures and potential confounders  25 young children who were infected with EV71 genotype C2, B4, C4, B5, and C4 in 1998, 2000, 2005, 2008 and 2010… |
|  |  | (b) Indicate number of participants with missing data for each variable of interest **-** |
| Outcome data | 15* | Report numbers of outcome events or summary measures  Neutralizing antibody titers were log transformed to calculate the geometric mean titers (GMTs), and their 95% confidence intervals (95% CI). The GMTs of cross-reactive neutralizing antibody titers were further used to generate an antigenic map using a web-based analytical tool. The relative positions of strains and antisera were adjusted such that the distances between strains and antisera in the map represent the corresponding ratios between homologous and heterologous neutralizing antibody titers. |
| Main results | 16 | (*a*) Give unadjusted estimates and, if applicable, confounder-adjusted estimates and their precision (eg, 95% confidence interval). Make clear which confounders were adjusted for and why they were included  children infected with genotype C2, C4, B4 and B5 had lower GMTs (>4-fold difference) against genotype A than other genotypes. In contrast, antigenic variations between genogroup B and C did not have a clear pattern…  Overall, genotypes in genogroup B and C clustered together and genotype A was found to be outside of the cluster  the nucleotide differences in the P1 region within genogroup were much lower than that between genogroups (0.042~0.151 vs. 0.188~0.235) but the differences in amino acid sequences were not as abundant as found in nucleotide sequences…  P1 regions were aligned to reveal that five amino acid signatures (N143D in VP2; K18R, H116Y, D167E, and S275A in VP1) are specific for genogroup A and may be related to the observed antigenic variations |
|  |  | (*b*) Report category boundaries when continuous variables were categorized  **-not applicable** |
|  |  | (*c*) If relevant, consider translating estimates of relative risk into absolute risk for a meaningful time period  **-not applicable** |
| Other analyses | 17 | Report other analyses done—eg analyses of subgroups and interactions, and sensitivity analyses  **-not applicable** |
| Discussion | | |
| Key results | 18 | Summarise key results with reference to study objectives  In our study, we found that children infected with genotype C2, C4, B4 and B5 had lower GMTs (≧4-fold difference) against genotype A than other genotypes but antigenic variations between genogroup B and C did not have a clear pattern… |
| Limitations | 19 | Discuss limitations of the study, taking into account sources of potential bias or imprecision. Discuss both direction and magnitude of any potential bias  Most clinical studies, including our study faced the limitation of small sample size due to the difficulty of collecting large amounts of serum samples from young children.  It is hard to compare different studies which employed different human sera and laboratory procedures, in particular the cell lines and virus strains used in the neutralization assay. |
| Interpretation | 20 | Give a cautious overall interpretation of results considering objectives, limitations, multiplicity of analyses, results from similar studies, and other relevant evidence  primary infection of genotype B5, two studies detected partial antigenic differences between genogroup B and C…  Kung *et al.* did not detect significant antigenic differences between genotypes B4 and C4 viruses using acute-phase sera…  A serological survey in healthy Japanese children and adults detected partial antigenic differences between genotype B5 and A viruses but not among different genotypes in genogroup B and C…  In a monkey study, Arita et al. found that… |
| Generalisability | 21 | Discuss the generalisability (external validity) of the study results  Based on the cross-reactive neutralizing antibody presented in the current study, genogroup B and C viruses are expected to induce protective neutralizing antibodies against genogroup B and C viruses but not genogroup A viruses.  we combined human serological data and viral genetic sequence data to identify five amino acid positions (4 on VP1 protein and 1 on VP2 protein) related to antigenic variations. Only one of these five positions (VP2 143) was also identified in the mice monoclonal antibody studies  Our study also found that the phylogenetic trees based on VP1 and P1 nucleotide sequences differ slightly |
| Other information | | |
| Funding | 22 | Give the source of funding and the role of the funders for the present study and, if applicable, for the original study on which the present article is based  This study was funded by the National Science Council, Taiwan (NSC 99-2811-B-002-180, 99-2321-B-002-025) and National Health Research Institutes, Taiwan. The funding organization had no role in the design and conduct of the study; collection, management, analysis, and interpretation of the data; or the preparation, review, or approval of the manuscript. |

*Give information separately for exposed and unexposed groups.

**Note:** An Explanation and Elaboration article discusses each checklist item and gives methodological background and published examples of transparent reporting. The STROBE checklist is best used in conjunction with this article (freely available on the Web sites of PLoS Medicine at http://www.plosmedicine.org/, Annals of Internal Medicine at http://www.annals.org/, and Epidemiology at http://www.epidem.com/). Information on the STROBE Initiative is available at www.strobe-statement.org.
